# Nexplanon migration into a subsegmental branch of the pulmonary artery: A case report and review of the literature

**DOI:** 10.1097/MD.0000000000018881

**Published:** 2020-01-24

**Authors:** Joya-Rita Hindy, Tarek Souaid, Corinne Tuckey Larus, Joanne Glanville, Ramzi Aboujaoude

**Affiliations:** a Faculty of Medicine, Saint Joseph University, Beirut, Lebanon; b Department of OB-GYN; c Department of General Surgery at Johnston-Willis Hospital, Richmond, VA, USA.

**Keywords:** contraceptive, embolism, implant, migration, Nexplanon

## Abstract

**Introduction:**

Nexplanon is a 4 cm rod-shaped barium sulphate coated contraceptive implant with a usual subdermal insertion in the inner non-dominant upper arm. Complications proper to subdermal contraceptive implants are unusual and principally localized and minor, comprising infection at the site of implantation, hematoma, abnormal scar development, or local nerve and blood vessel injuries. Infrequently, contraceptive implant migration can happen, though habitually not far from the site of insertion. Pulmonary embolization of the device is remarkably rare and can present with symptoms such as chest pain or dyspnea.

**Patient concerns and diagnosis:**

We report one of the rare cases of asymptomatic Nexplanon pulmonary embolism in a 26-year-old female.

**Interventions and outcomes:**

An endovascular intervention successfully retrieved the device from the lateral segment right middle lobe pulmonary artery without any complications.

**Conclusion:**

Several cases of contraceptive implant migration into the pulmonary artery have been reported to this day. Preventing this life-threatening complication is challenging, and yet, no clear guidelines have been established.

## Abstract 2

Supplemental Digital Content is available in the text

## 1 Introduction

Since their introduction in the United States in 1991, subdermal implants have become safe and efficient widespread contraceptive options.^[1]^ Nexplanon (Merck Whitehouse Station, NJ) is an implant that offers 450 pg/mL for initial serum level of etonogestrel, the active metabolite of desogestrel, with a steady decrease to nearly 200 pg/mL at the end of the third year.^[2]^ It has a Pearl index of 0.0 (95% confidence interval, 0.0–0.2), making it a highly effective contraceptive method for a period of 2 to 3 years.^[3]^ It is a 4 cm rod-shaped contraceptive implant with a usual subdermal insertion in the inner non-dominant upper arm. Its barium sulphate coat makes it radiopaque, therefore detectable on X-ray or Computed Tomography (CT).^[4]^ Complications proper to subdermal contraceptive implants are unusual and principally localized and minor, comprising infection at the site of implantation, hematoma, abnormal scar development, or local nerve and blood vessel injuries.^[5]^ Nexplanon extraction is performed in the outpatient setting through a small incision of the overlying skin. Infrequently, contraceptive implant migration can happen, though habitually not far from the site of insertion.^[6]^ Pulmonary embolization of the device is remarkably rare and can present with symptoms such as chest pain or dyspnea.^[7]^ We report one of the rare cases of asymptomatic Nexplanon pulmonary embolism in a 26-year-old female, from implantation to extraction.

## 2 Case report

On October 2015, a 26-year-old Caucasian female, G0P0, with a Body Mass Index (BMI) of 20.63, requested a Nexplanon implantation for contraceptive coverage. The device was placed in the left arm during a local procedure without any subsequent complication.

During the 3 following years, she reported no significant complains and did not present with any possible side effects of the device.

On July 2018, 3 years after the implantation of the device, she requested the Nexplanon removal. The device was not palpable under her left arm skin; therefore, the standard removal procedure could not be done.

**Figure 1:**
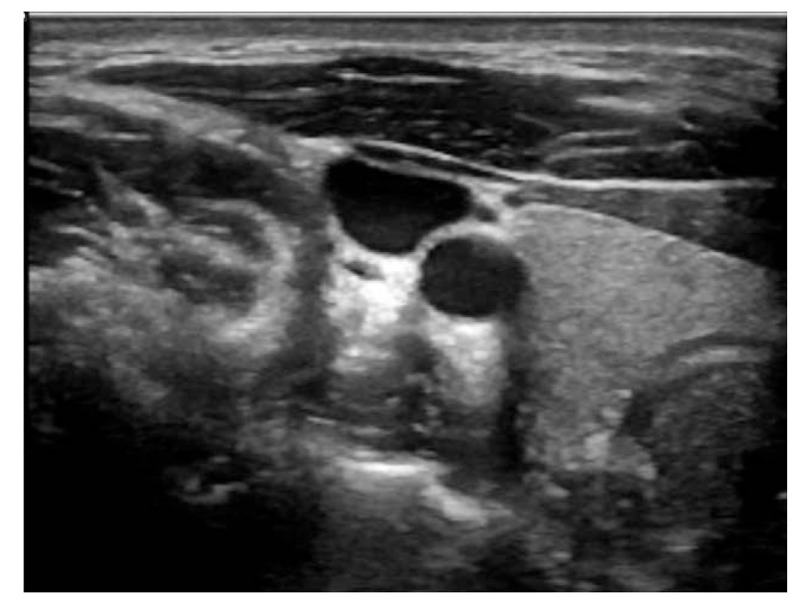
Intraoperative ultrasound of left arm showing no Nexplanon.

On August 2018, an intraoperative ultrasound and fluoroscopy of the left arm (from the left axillary area to the left elbow) (Fig. 1) and the left chest were done and no Nexplanon was identified.

**Figure 2:**
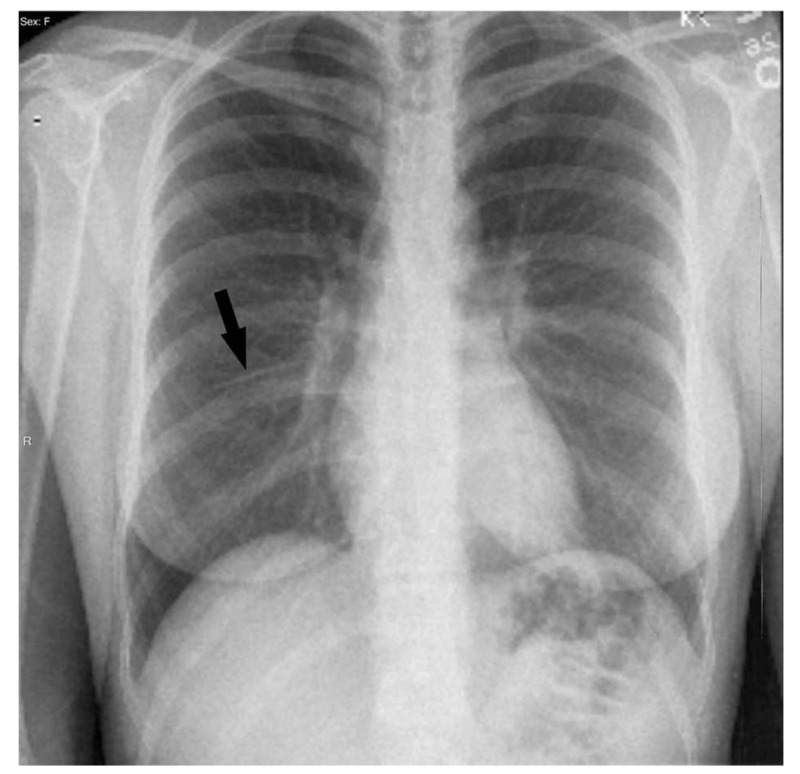
Chest X-ray showing the Nexplanon (arrow) projecting over the right perihilar lung.

Subsequently, a chest X-ray was performed and found an elongated foreign body projecting over the right perihilar lung suspicious for migrated contraceptive implant (Fig. 2). No pneumothorax or pleural effusion was apparent and the cardiomediastinum was unremarkable.

**Figure 3:**
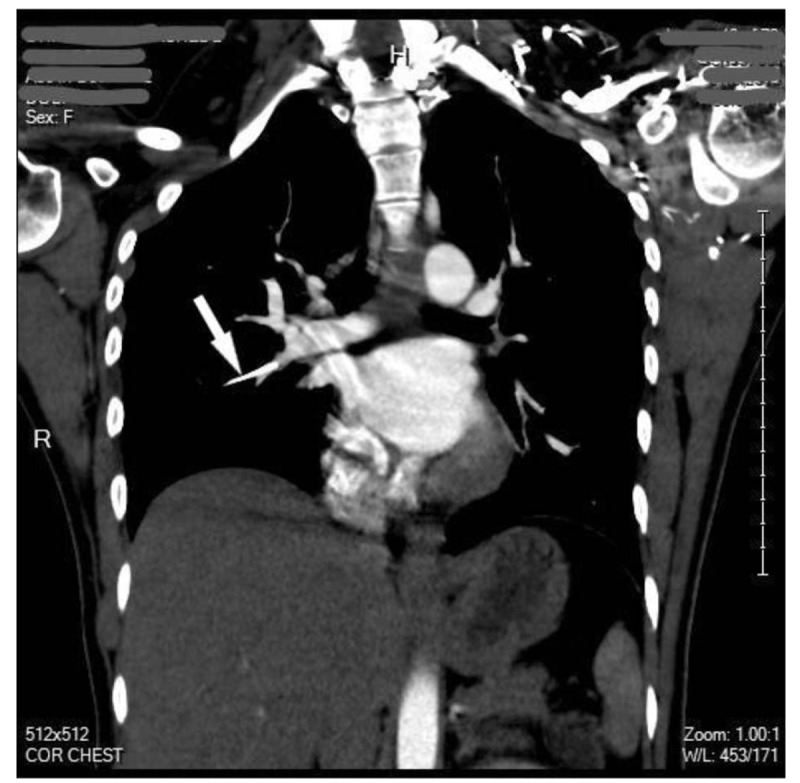
CT chest angiography showing the migrated Nexplanon (arrow) in the right middle lobe pulmonary artery.

At last, the patient underwent a CT chest angiography. It showed that the Nexplanon initially implanted in the left upper extremity has migrated to the right middle lobe pulmonary artery (Fig. 3).

**Figure 4:**
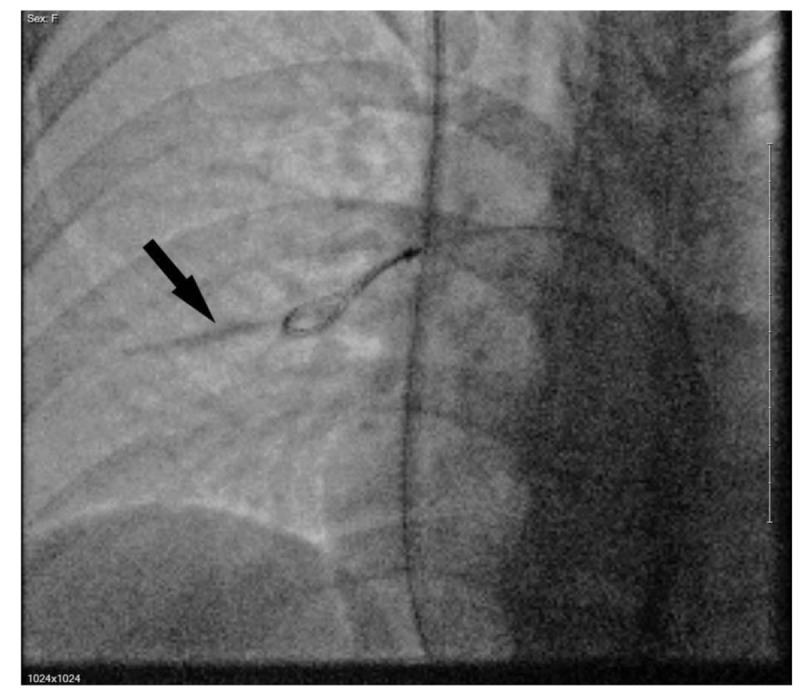
Pulmonary arteriography (right internal jugular vein access) showing fully intact contraceptive device (arrow) just before retrieval by an Ensnare loop.

On September 2018, the embolized contraceptive device was found in the lateral segment right middle lobe pulmonary artery. The implant was then removed via a pulmonary arteriography with right internal jugular vein access (Fig. 4). The fully intact device was retrieved by an Ensnare loop (see supplemental video [Video illustrating a pulmonary arteriography with right internal jugular vein access retrieving the intact device by an Ensnare loop, 22 s, 53.8 MB]) with no immediate complications.

## 3 Discussion and conclusion

The usual medical complications associated with contraceptive implants are menstrual disturbances, headache, weight gain, acne, dizziness, mood disturbances, nausea, lower abdominal pain, hair loss, loss of libido, pain at the implant site, neuropathy, and follicular cysts.^[5]^ None of the above were seen in this reported case.

Subdermal contraceptive implant embolism to the lungs and the pulmonary arteries is an emerging iatrogenic condition. In fact, 11 previously published cases in the PubMed literature have been summarized (see Table 1). In addition, a query of the Food and Drug Administration (FDA)'s Adverse Event Reporting System (FAERS) database until 2015 revealed 38 patients with etonogestrel implant migration, from which, 9 cases were in the lung or pulmonary artery.^[8]^ Some of them did have some shortness of breath and chest pain/discomfort. Therefore, at least 21 cases (including this report) of contraceptive implant pulmonary embolization have been reported to date; raising awareness for the prevention/detection of this life-threatening complication may help reduce the risks.

**Table 1:**
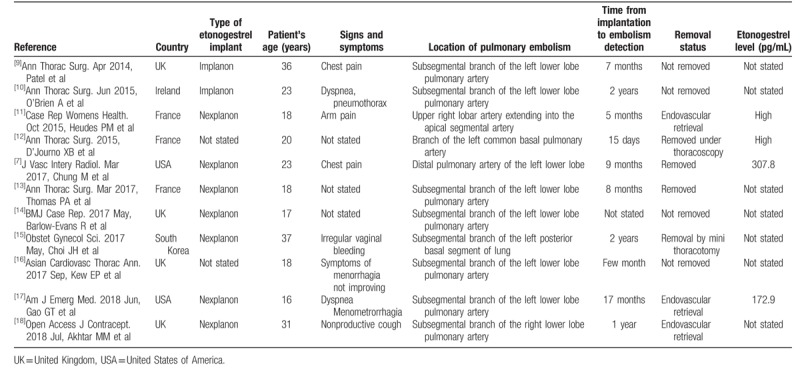
Summary of 11 previously published cases of pulmonary embolism due to contraceptive implant migration.

When the implant is not palpable under the arm's skin, it should be directly located. Therefore, an arm ultrasound or X-ray should be acquired as a standard workup. If these tests fail to show the presence of the implant (as a radiopaque or hyperechoic line), it is important to consider a more distant migration. Thus, a chest radiograph and furthermore a CT angiography should be performed. An etonogestrel level can also be used to confirm or deny the presence of the device.

After a contraceptive implant migration in pulmonary artery, there may be serious cardiopulmonary complications such as infection, further migration and thrombosis. It is crucial to determine the exact location of the implant as endovascular interventions will be used to retrieve it with high success rate and low morbidity.

The exact mechanism by which the implant may embolize is unclear. An inadvertent placement of the Nexplanon into the venous system or into the deep subcutaneous tissue may occur during the initial procedure. And consequently, a migration to the pulmonary vasculature through the right side of the heart may occur. It could be hypothesized that the lack of subcutaneous tissue may be a potential risk factor for the device's migration. Thus, it could be useful to seek a correlation with the cases’ BMI. However, most cases reported do not indicate patients’ BMI.

Subdermal contraceptive implant insertion is complex, and attention should be drawn into this to operate carefully. According to the instructions for insertion, the Nexplanon should be placed subdermally at the inner side of the upper nondominant arm about 7 cm above the elbow crease in the groove between the biceps and the triceps. If the recommendations are followed, it is unlikely to go beyond 1 cm in depth, especially that the Nexplanon is usually implanted more superficially than the Implanon device. Moreover, it is often unclear if migration happened at the time of insertion or at a considerable distance in time.

The challenge when it comes to this topic is to be able to detect device migration as soon as it occurs. A self-examination and a follow-up with the physician may be possible solutions and useful trackers of a potential migration. The frequency and technique of self-examination and follow-up better be determined and systematized by experts.

## Author contributions

**Conceptualization:** Joya-Rita Hindy, Tarek Souaid, Ramzi Aboujaoude.

**Resources:** Corinne Tuckey-Larus, Joanne Glanville.

**Supervision:** Corinne Tuckey-Larus, Joanne Glanville, Ramzi Aboujaoude.

**Writing – original draft:** Joya-Rita Hindy, Tarek Souaid.

**Writing – review & editing:** Ramzi Aboujaoude.

## Supplementary Material

Supplemental Digital Content
